# Idiopathic Syringomyelia: Diagnostic Value of Cranial Morphometric Parameters

**DOI:** 10.3390/brainsci15080811

**Published:** 2025-07-29

**Authors:** Birol Özkal, Hakan Özçelik

**Affiliations:** Department of Neurosurgery, Alanya Alaaddin Keykubat University, Alanya 07400, Türkiye; hakan.ozcelik@alanya.edu.tr

**Keywords:** idiopathic syringomyelia, Chiari Type 0, Chiari Type I, posterior fossa, morphometric analysis, magnetic resonance imaging

## Abstract

**Background:** Identifying the etiological factors of syringomyelia, which can cause progressive neurological deficits in the spinal cord, is critically important for both diagnosis and treatment. This study aimed to assess the cranial morphometric features of patients with idiopathic syringomyelia by conducting comparative analyses with individuals diagnosed with Chiari Type I, Chiari Type I accompanied by syringomyelia, and healthy controls, in order to elucidate the potential structural contributors to the pathogenesis of idiopathic syringomyelia. **Methods:** In this retrospective and comparative study, a total of 172 patients diagnosed with Chiari Type I and/or syringomyelia between 2016 and 2024, along with 156 radiologically normal individuals, were included. The participants were categorized into four groups: healthy controls, Chiari Type I, Chiari Type I with syringomyelia, and idiopathic syringomyelia (defined as syringomyelia without an identifiable cause). Midline sagittal T1-weighted MR images were used to obtain quantitative measurements of the posterior fossa, cerebellum, intracranial area, and foramen magnum. All measurements were stratified and statistically analyzed by sex. **Results:** In cases with idiopathic syringomyelia, both the posterior fossa area and the cerebellum/posterior fossa ratio differed significantly from those of healthy controls. In male patients, the foramen magnum diameter was significantly larger in the Chiari + syringomyelia group compared with the idiopathic group. A significant correlation was found between the degree of tonsillar descent and selected morphometric parameters in female subjects, whereas no such correlation was observed in males. Both Chiari groups exhibited significantly smaller posterior fossa dimensions compared with the healthy and idiopathic groups, indicating greater neural crowding. Additionally, in Chiari Type I patients, increasing degrees of tonsillar descent were associated with a decreased incidence of syringomyelia. **Conclusions:** Anatomical variations such as a reduced posterior fossa area or altered foramen magnum diameter may contribute to the pathogenesis of idiopathic syringomyelia. Cranial morphometric analysis appears to offer diagnostic value in these cases. Further prospective, multicenter studies incorporating advanced neuroimaging modalities, particularly those assessing cerebrospinal fluid dynamics, are warranted to better understand the mechanisms underlying syringomyelia of unknown etiology.

## 1. Introduction

Syringomyelia (SM) is a rare neurological disorder characterized by the formation of fluid-filled cystic cavities (syrinx) within the spinal cord. In the general population, its prevalence is estimated at approximately 8–10 cases per 100,000 individuals [1]. SM commonly develops secondary conditions such as Chiari Type I (CM-1) malformation, spinal trauma, tumors, or infections. However, in some cases, no structural cause can be identified; such instances are classified as “idiopathic syringomyelia”. Etiology-based management strategies are generally more effective than symptomatic treatments and are associated with reduced morbidity and mortality rates, leading to more favorable clinical outcomes [2,3].

Recent studies have highlighted the significance of anatomical parameters—such as posterior fossa volume, cerebellar area, and foramen magnum diameter—in the pathogenesis of CM-1 malformation, which typically involves the herniation of the cerebellar tonsils through the foramen magnum [4]. The incidence of SM in patients with CM-1 has been reported to be as high as 50% [5]. However, the fact that SM does not develop in all Chiari cases suggests the presence of other, yet unidentified, intracranial morphometric determinants.

Morphometric alterations such as a reduced posterior fossa size, decreased cisterna magna volume, and angular anomalies of the brainstem have been shown to mechanically restrict cerebrospinal fluid (CSF) flow at the level of the foramen magnum. This restriction may result in pressure gradients and turbulent flow patterns. Such hydrodynamic disturbances can create a predisposed environment for syrinx formation, even in the absence of overt tonsillar herniation. Phase-contrast magnetic resonance imaging (MRI) studies have demonstrated that individuals with a small posterior fossa volume may exhibit abnormal CSF flow patterns at the craniovertebral junction, including delayed or diminished systolic and diastolic flow velocities [5,6,7,8,9,10]. Cases exhibiting Chiari-like symptoms and SM in the absence of significant tonsillar descent have been defined as “Chiari Type 0” (CM-0) In these patients, posterior fossa crowding is thought to play a key role [10]. These findings support the hypothesis that morphometric constraints within the posterior cranial fossa may directly impair CSF circulation and contribute to syrinx formation, particularly in idiopathic SM cases.

The aim of this study is to evaluate the morphometric features of the posterior fossa and cerebellar structures in individuals with idiopathic SM who do not exhibit cerebellar tonsil herniation and have no identifiable etiological factor. Additionally, by comparing these individuals with healthy controls and those with Chiari Type I-associated SM, this study seeks to identify morphometric parameters that may have diagnostic and etiological significance, with a particular focus on their distinction from CM-0 cases.

## 2. Materials and Methods

### 2.1. Study Design and Participants

This retrospective, observational, and comparative study was conducted using patient data from the Department of Neurosurgery at Alanya Alaaddin Keykubat University, between 2016 and 2024. The study group included patients diagnosed with Arnold Chiari malformation and/or SM, while the control group consisted of individuals with normal brain and spinal MRI findings. Data were retrieved from the institutional hospital information system.

### 2.2. Inclusion and Exclusion Criteria

Individuals aged between 18 and 60 years with complete and high-quality MRI scans of the brain and spinal cord were included. CM-1 diagnosis was based on cerebellar tonsil herniation of ≥5 mm below the foramen magnum [11]. SM was diagnosed when a centrally located, CSF-isointense intramedullary cyst ≥2 mm in diameter was observed on T2-weighted images [1]. The control group was composed of subjects who underwent brain and spinal MRI during the same period and were found to have no pathological findings. These individuals were randomly selected among eligible cases, ensuring age and sex compatibility across groups.

In idiopathic SM cases, individuals presenting with brainstem-related symptoms commonly associated with CM-1 (such as headache, dysphagia, dysarthria, and respiratory disturbances) were excluded from the study in order to rule out the possibility of CM-0.

Across all groups, the following conditions were defined as exclusion criteria: spinal deformities, spina bifida, tethered cord, a history of spinal trauma, infections such as arachnoiditis, severe discopathy, spinal stenosis, multiple sclerosis or other neurodegenerative diseases, hydrocephalus, prior spinal surgery, craniovertebral junction anomalies, intramedullary tumors, intracranial mass lesions, pathologies associated with altered intracranial pressure (e.g., pseudotumor cerebri), endocrine or metabolic disorders affecting CSF circulation, the presence of severe systemic disease, metallic implants or devices that may cause artifacts during MRI, and inadequate image quality [12,13].

### 2.3. Data Collection and Imaging Parameters

All subjects underwent analysis of mid-sagittal T1-weighted brain MRI, the standard imaging modality for assessing Chiari malformations [4,14,15,16]. The scans were acquired on a 1.5 Tesla Siemens Magnetom Altea scanner (Erlangen, Germany).

All MRI images were manually reviewed and analyzed using Sectra IDS7 Workstation (Version 24.2.16.6066, 2023). Morphometric measurements were obtained from the largest mid-sagittal slice, defined as the image section clearly displaying the foramen magnum, brainstem, fourth ventricle, and cerebellar tonsils in maximal anatomical extent.

All morphometric measurements were independently performed by two board-certified neurosurgeons who were blinded to the clinical group assignments. To evaluate inter-rater reliability, the Intraclass Correlation Coefficient [ICC(2,1)] was calculated for each morphometric parameter using a two-way random-effects model with absolute agreement. This model assumes that raters are randomly sampled from a larger population and evaluates the degree of exact agreement between measurements. The ICC values ranged from 0.87 to 0.96, indicating excellent reliability between the two raters [17].

Morphometric measurements were obtained from the largest sagittal slice of each structure, including the foramen magnum, tonsillar herniation, cerebellum, posterior fossa, and intracranial area.

Imaging Parameters:

- T1-weighted: TR 250 ms, TE 4.76 ms.
- T2-weighted: TR 3000–4000 ms, TE 100–120 ms.
- Slice thickness: 3 mm, inter-slice gap: 0.5 mm.
- Field of view: 230–280 mm.
- Matrix: 205 × 256 (T1) and 320 × 320 (T2).

All images were manually reviewed and evaluated by board-certified neurosurgeons using a Sectra IDS7 Workstation (Edition 24.2.16.6066, 2023).

**Figure 1:**
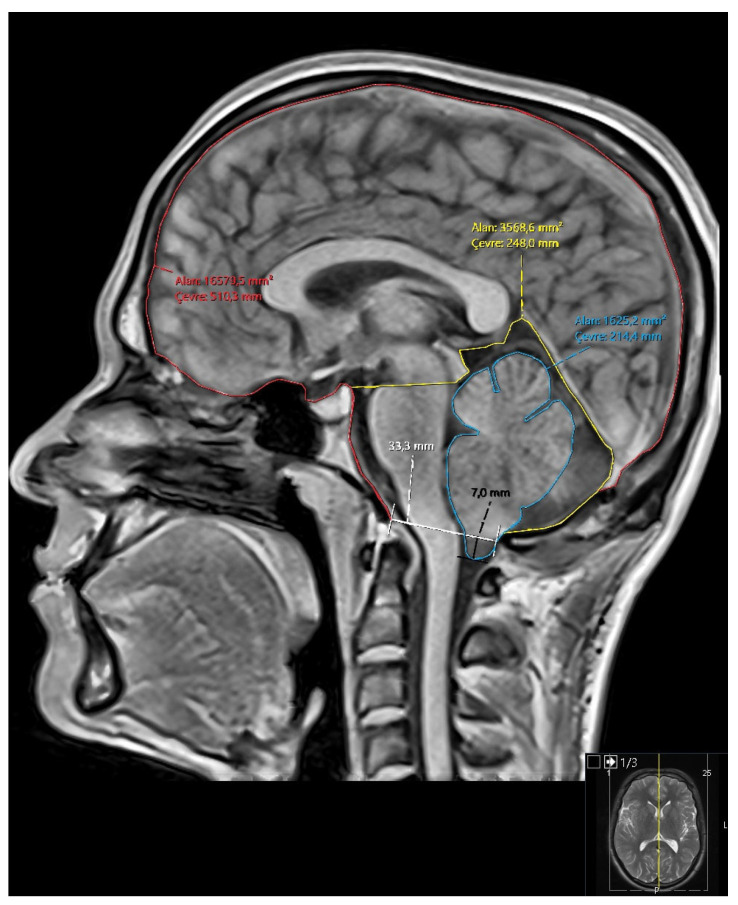
Representative mid-sagittal T1-weighted brain MRI demonstrating cranial morphometric measurements used in the study. Traced regions include the intracranial area (red), posterior fossa (yellow), and cerebellum (blue). Quantitative measurements include area and perimeter of each region, foramen magnum diameter (white), and degree of cerebellar tonsillar descent (black).

Morphometric Parameters Measured:

- Diameter of the foramen magnum.
- Degree of tonsillar descent (for Groups 2 and 3).
- Area and perimeter of the posterior fossa.
- Area and perimeter of the cerebellum.
- Area and perimeter of the intracranial cavity.
- Ratios: cerebellum/posterior fossa, posterior fossa/intracranial area, cerebellum/intracranial area (Figure 1).

In SM cases, lesion location (cervical, thoracic, etc.), size, and longitudinal extent were recorded in three planes. Contrast-enhanced MRI was performed in selected cases to rule out accompanying pathologies.

### 2.4. Group Classification

- **Group 1 (Control):** Healthy individuals with no pathological findings on brain or spinal MRI.
- **Group 2 (Chiari):** Patients with ≥5 mm cerebellar tonsil herniation.
- **Group 3 (Chiari + SM):** Patients with both tonsillar herniation and SM.
- **Group 4 (Idiopathic SM):** Patients who met criteria for idiopathic SM.

### 2.5. Statistical Analysis

All statistical analyses were conducted using R statistical software (R Core Team, Vienna, Austria, 2024). Prior to the main analyses, a comprehensive data cleaning process was performed. First, missing data were examined, and for variables with less than 5% missingness, listwise deletion was applied. Subsequently, outlier analysis was carried out. Values with z-scores > ±3.29 were considered potential univariate outliers, while boxplots and the Mahalanobis distance were used to detect multivariate outliers. Observations confirmed as outliers were excluded from further analysis.

Following data cleaning, the distribution of all continuous variables was assessed using the Kolmogorov–Smirnov test. Variables with a normal distribution were analyzed using one-way ANOVA, whereas non-normally distributed variables were assessed using the Kruskal–Wallis test. Where significant group differences were identified, post hoc analyses were conducted accordingly.

To control for Type I error due to multiple comparisons, appropriate correction methods were applied. For ANOVA-based comparisons, the Bonferroni correction was used. However, depending on the number of comparisons and distributional characteristics, the Benjamini–Hochberg False Discovery Rate (FDR) correction was also considered and applied when deemed more appropriate. For non-parametric comparisons, the Mann–Whitney U test was employed along with the Bonferroni adjustment. This combined approach ensured statistical rigor while minimizing the risk of false positives.

Although efforts were made to maintain balanced group sizes, the idiopathic SM group was relatively smaller than the others due to the lower prevalence of this condition. This imbalance was considered during statistical planning, and non-parametric tests (e.g., Kruskal–Wallis, Mann–Whitney U) were applied in cases where assumptions of homogeneity of variance or normality were violated. Additionally, results from smaller groups were interpreted with caution, particularly when marginal *p*-values or large standard deviations were observed.

A priori power analysis was conducted using G*Power 3.1 software to evaluate whether the study had a sufficient sample size to detect meaningful effects [18]. Based on a one-way ANOVA framework with a significance level of α = 0.05, a medium effect size (Cohen’s f = 0.25), and four group comparisons, the analysis yielded a statistical power (1 − β) of ≥0.90. This indicates that the study had adequate sensitivity to detect the expected effect sizes with a high level of confidence.

Associations between the degree of tonsillar descent and cranial morphometric parameters were assessed using Spearman’s rank correlation coefficient, due to the non-parametric nature of the variables.

Given known anatomical differences between sexes, all morphometric comparisons were stratified by gender and supported by detailed descriptive statistics (mean, standard deviation, median, and range).

## 3. Results

### 3.1. Participants

A total of 328 individuals were included in the study as follows: 156 healthy controls (Group 1), consisting of 93 females (59%) and 63 males (41%); 79 patients with CM-1 (Group 2), including 54 females (68%) and 25 males (32%); 39 patients with Chiari + SM (Group 3), including 25 females (64%) and 14 males (36%); and 54 patients with idiopathic SM (Group 4), including 30 females (55%) and 24 males (45%).

The mean ages of the male participants were:

Group 1: 36.83 years (range: 18–60).
Group 2: 34.08 years (19–60).
Group 3: 42.36 years (24–60).
Group 4: 41.08 years (18–60).

**Figure 2:**
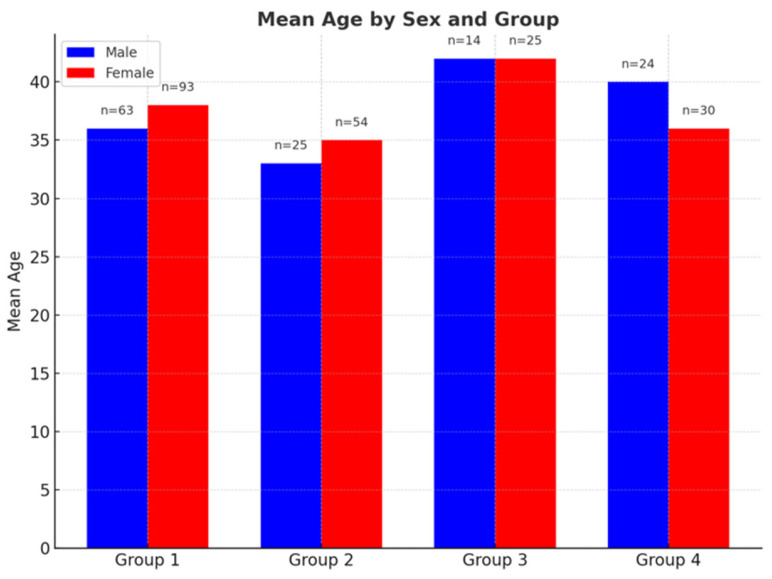
Mean age by sex across four participant groups. Blue bars represent male participants and red bars represent female participants. Sample sizes (*n*) are indicated above each bar. While mean ages are generally similar between sexes within each group, Group 3 displays the highest mean age values for both males and females.

The mean ages of the female participants were:

Group 1: 37.97 years (range: 18–60).
Group 2: 38.74 years (18–60).
Group 3: 42.84 years (23–60).
Group 4: 34.57 years (18–60) (Figure 2).

### 3.2. Morphometric Analysis in Female Participants

**Figure 3:**
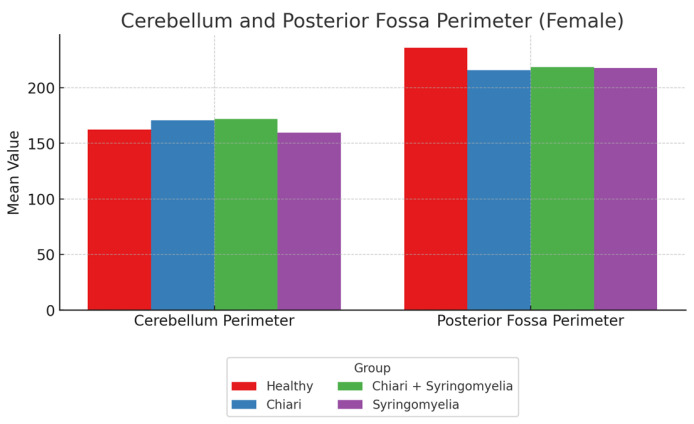
Mean values of cerebellum perimeter and posterior fossa perimeter in four female subgroups: healthy (*n* = 93), Chiari (*n* = 54), Chiari with SM (*n* = 25), and idiopathic SM (*n* = 30). Posterior fossa perimeter was highest in the healthy group, whereas cerebellum perimeter was greater in the Chiari and Chiari + SM groups.

**Figure 4:**
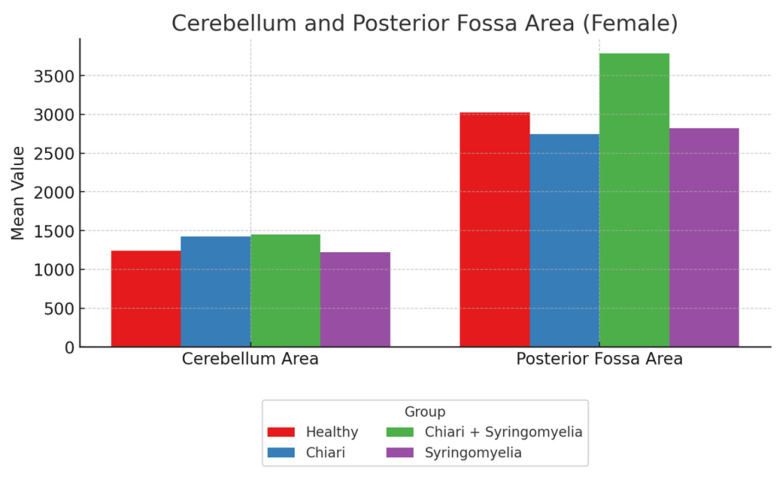
Comparison of cerebellum area and posterior fossa area measurements across four female subgroups: healthy (*n* = 93), Chiari (*n* = 54), Chiari with SM (*n* = 25), and idiopathic SM (*n* = 30). Data represent mean values. The Chiari + SM group showed the highest posterior fossa area, while cerebellum area varied less across groups.

No statistically significant differences were found among the groups in terms of foramen magnum diameter and intracranial area, or its perimeter (*p* > 0.05). The posterior fossa perimeter was significantly higher in Group 1 compared with Groups 2, 3, and 4 (*p* < 0.001). The cerebellar perimeter was significantly larger in Groups 2 and 3 compared with Group 4 (*p* < 0.001) (Figure 3). The posterior fossa area was significantly larger in Groups 1, 2, and 3 compared with Group 4 (*p* < 0.05), and Group 3 had a significantly larger posterior fossa area than Group 2 (*p* < 0.05). The cerebellum area was significantly greater in Groups 2 and 3 than in Groups 1 and 4 (*p* < 0.05) (Figure 4).

**Figure 5:**
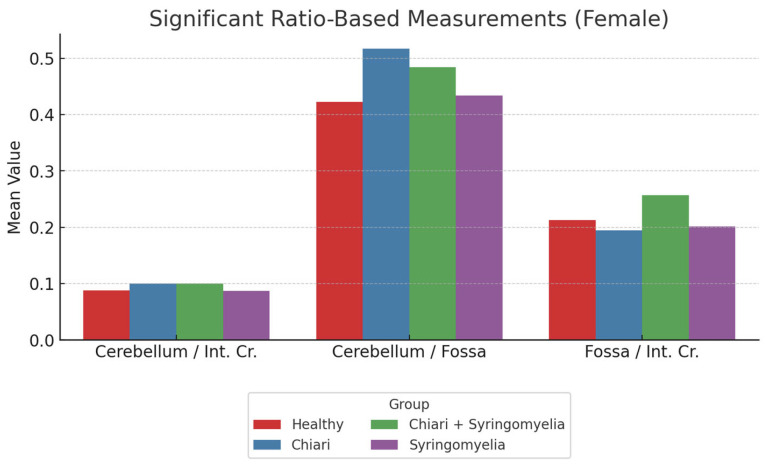
Comparison of ratio-based morphometric values among female subgroups: healthy (*n* = 93), Chiari (*n* = 54), Chiari with SM (*n* = 25), and idiopathic SM (*n* = 30). The graph illustrates three key ratios: cerebellum/brain, cerebellum/fossa, and fossa/brain. The Chiari group showed the highest cerebellum/fossa ratio, while cerebellum/brain ratio peaked in the Chiari + SM group. Cerebellum:cerebellum area, Int. Cr.—intracranial area, Fossa—posterior fossa area.

The ratio analyses revealed the following:

- The posterior fossa/intracranial area ratio was higher in Group 1 than in Groups 2 and 3; and higher in Group 3 than Group 2 (*p* < 0.05).
- The cerebellum/posterior fossa ratio was significantly higher in Groups 2 and 3 compared with Group 1 (*p* < 0.05).
- The cerebellum/intracranial area ratio was significantly higher in Groups 2 and 3 than in Groups 1 and 4 (*p* < 0.05) (Figure 5, Table 1).

### 3.3. Morphometric Analysis in Male Participants

**Figure 6:**
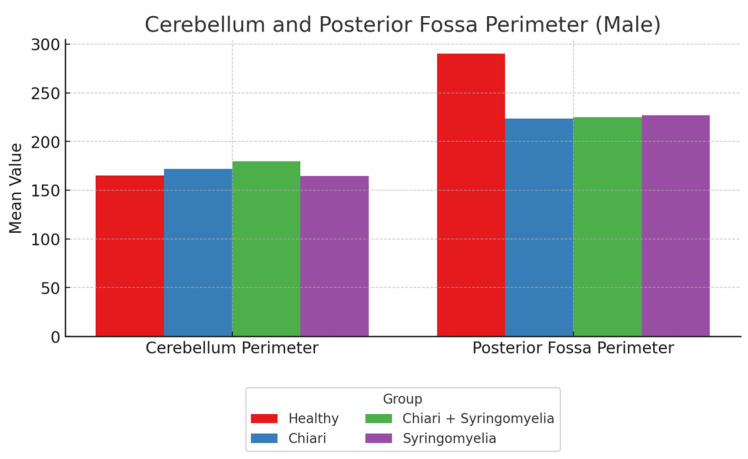
Group-wise comparison of cerebellum and posterior fossa perimeter among male participants. The healthy group exhibited the highest posterior fossa perimeter, while the cerebellum perimeter was greater in the Chiari and Chiari + SM subgroups. Healthy (*n* = 63), Chiari (*n* = 25), Chiari with SM (*n* = 14), and idiopathic SM (*n* = 24).

**Figure 7:**
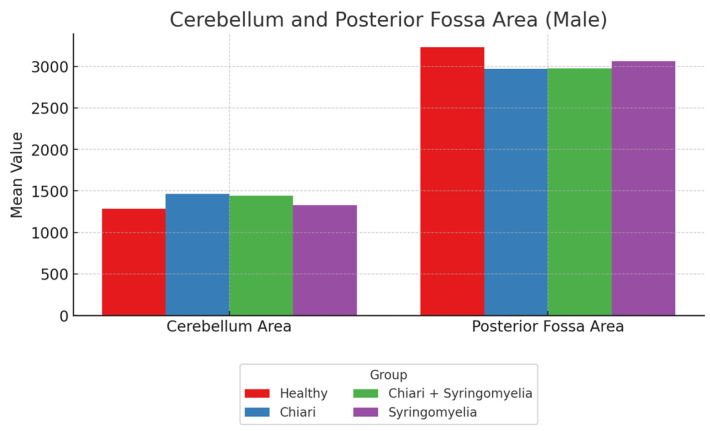
Mean values of cerebellum area and posterior fossa area among male subgroups: healthy (*n* = 63), Chiari (*n* = 25), Chiari with SM (*n* = 14), and idiopathic SM (*n* = 24). While cerebellum area was relatively consistent across groups, posterior fossa area showed slightly higher values in the healthy and SM groups.

No significant differences were observed in the intracranial area and perimeter parameters among the groups. The foramen magnum diameter was significantly larger in Group 3 compared with Group 4 (*p* < 0.05). The cerebellar perimeter was significantly greater in Group 3 compared with Groups 1 and 4 (*p* < 0.05) (Figure 6). Regarding cerebellum area, Group 2 showed significantly larger values than Groups 1 and 4; Group 3 was significantly larger than Group 1 (*p* < 0.05). The posterior fossa perimeter and area were significantly higher in Group 1 than all other groups (*p* < 0.05) (Figure 7).

**Figure 8:**
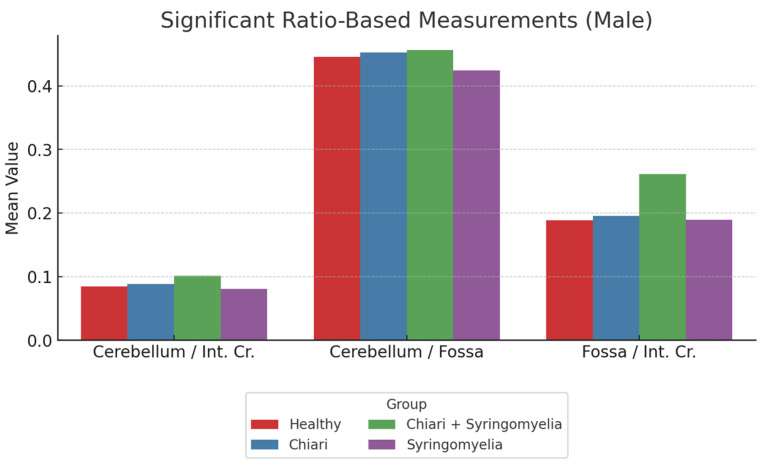
Comparison of cerebellum/brain, cerebellum/fossa, and fossa/brain ratios among male subgroups. The Chiari + SM group demonstrated the highest cerebellum/brain and fossa/brain ratios, whereas cerebellum/fossa ratio peaked in the Chiari group. Healthy (*n* = 63), Chiari (*n* = 25), Chiari with SM (*n* = 14), and idiopathic SM (*n* = 24). Cerebellum: Cerebellum area, Int. Cr.—Intracranial area, Fossa—Posterior fossa area.

A further ratio analysis showed the following:

- The cerebellum/posterior fossa ratio was lowest in Group 1 and significantly higher in Groups 2 and 3 (*p* < 0.05).
- The posterior fossa/intracranial area ratio was significantly higher in Group 1 than all other groups; and in Group 4 compared with Group 2 (*p* < 0.05).
- The cerebellum/intracranial area ratio was significantly higher in Groups 2 and 3 than in Groups 1 and 4 (*p* < 0.05) (Figure 8, Table 2).

### 3.4. Tonsillar Herniation and Correlation Analysis

**Figure 9:**
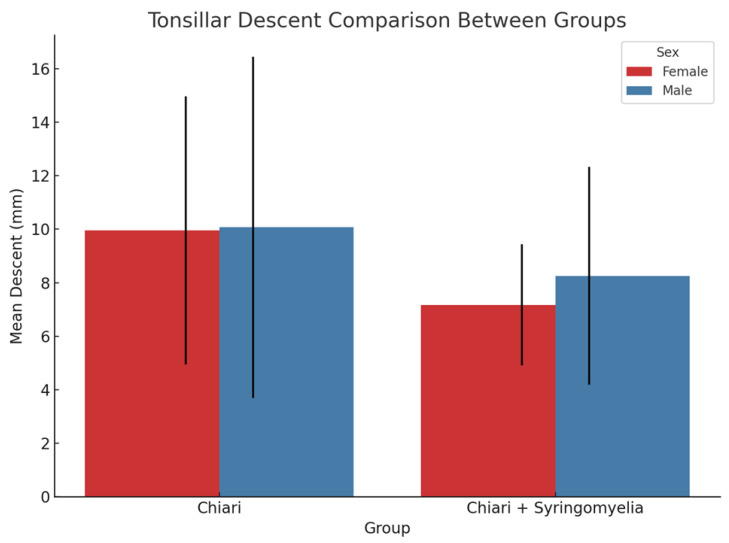
Comparison of mean tonsillar descent (in mm) between Chiari and Chiari + SM groups in male and female participants. Female participants in the Chiari group (*n* = 54) exhibited significantly greater tonsillar descent compared with those in the Chiari + SM group ( *n* = 25, *p* = 0.014). Although the male Chiari group showed a higher mean descent than the Chiari + SM group, the difference was not statistically significant (*p* = 0.403). Error bars represent standard deviation.

In both sexes, Group 2 showed significantly more tonsillar herniation than Group 3 (*p* < 0.05) (Figure 9). In female participants, a weak but significant positive correlation was found between tonsillar descent and both cerebellar perimeter (r = 0.372, *p* = 0.001) and cerebellum/posterior fossa ratio (r = 0.359, *p* = 0.001). No significant correlation was observed in male participants.

### 3.5. Syrinx Location and Distribution

In Group 3 (Chiari + SM), syrinx localization was as follows: 2 bulbar, 17 cervical, 9 cervicothoracic, 4 thoracic, 1 thoracolumbar, and 2 cervicothoracolumbar.

In Group 4 (idiopathic SM), 30 cervical, 9 cervicothoracic, 10 thoracic, and 3 thoracolumbar localizations were observed. No statistically significant difference in syrinx distribution was found between Group 3 and Group 4.

## 4. Discussion

Etiologically, approximately 50–70% of SM cases are associated with CM-1 malformation, 10–15% with spinal trauma, 5–10% with intramedullary tumors, and 2–5% with infections or arachnoiditis. The remaining 10–15% are considered idiopathic, with no identifiable structural pathology [16,19]. In the treatment of SM, etiologically targeted approaches are more effective in reducing mortality and morbidity, as they focus on addressing the underlying cause [2,3]. This leads to improved clinical outcomes and lower recurrence rates. In this context, our study aimed to investigate the possible etiological basis of cases classified as idiopathic SM.

CM-1 malformation is the most frequently observed cause of SM. It is defined by a ≥5 mm descent of the cerebellar tonsils through the foramen magnum due to the underdevelopment of the posterior fossa. This finding has high sensitivity and specificity when evaluated via MRI [4]. However, some studies have reported that not only tonsillar herniation but also posterior fossa morphometry plays a determining role in the diagnosis [20]. The exact morphometric basis for why only a subset of Chiari cases develop SM remains unclear.

The definition of CM-0 has revealed that Chiari I-like symptoms and SM can occur even in the absence of tonsillar herniation. In Chiari 0 cases, anatomical narrowing and CSF flow disturbances are thought to be independently determinative in the disease etiology. Therefore, cranial morphometric structures are suggested to play a significant role in the development of SM [10].

Intracranial structures undergo volumetric changes due to growth, degeneration, and aging. Previous studies suggest that measurements remain stable between the ages of 18 and 60, which defined our inclusion criteria [21,22].

It is well established that healthy males tend to have larger posterior fossa, cerebellar, and intracranial volumes, as well as a greater foramen magnum diameter, compared with females [10,23]. Accordingly, gender-based stratification is essential in morphometric assessments. The predominance of females in both our Chiari and SM groups aligns with prior studies showing a higher prevalence among women. The significant correlations observed in females are consistent with the findings suggesting that a lower posterior fossa volume and foramen magnum diameter in healthy women may predispose them to the development of CM-1 malformation and SM [24,25,26].

No significant differences were found between males and females in terms of intracranial area and perimeter, suggesting balanced group distributions and appropriate comparability.

In idiopathic SM cases presenting solely with SM and lacking Chiari I symptoms, it is notable that despite the absence of classic Chiari Type I findings, significant differences in posterior fossa and cerebellar volumes were observed. These findings support the notion that cranial morphometric structures may also play a role in cases classified as idiopathic.

In female participants, posterior fossa area and perimeter were significantly greater in healthy controls than in any of the pathological groups. This aligns with the literature suggesting a developmentally smaller posterior fossa in Chiari patients [2,10]. The larger cerebellar area and perimeter in Groups 2 and 3 compared with Group 4 may indicate that tonsillar descent is a response to posterior fossa crowding [5]. Studies of CM-0 cases have similarly shown that posterior fossa crowding, obex positioning, and cervical canal morphometry can be key diagnostic indicators [10,26], supporting our findings of morphometric changes in idiopathic cases even without tonsillar descent. However, the inverse relationship between the degree of tonsillar herniation and the presence of SM (Group 2 > Group 3) suggests that not only anatomical but also hydrodynamic factors may contribute to SM development.

The elevated cerebellum/posterior fossa and cerebellum/intracranial area ratios observed in the Chiari and idiopathic SM groups reflect cerebellar crowding within a compact posterior fossa and may serve as potential diagnostic parameters [27]. However, these ratios were lower in the idiopathic group than in the Chiari group, suggesting that cerebellar compression may be less pronounced in idiopathic cases [28].

In male participants, the findings largely mirrored those in females; Group 1 had significantly greater posterior fossa dimensions than all other groups. However, the differences in cerebellar area and ratios were more limited, possibly indicating greater compensatory volume capacity in males [21]. The significantly larger foramen magnum diameter in Group 3 compared with Group 4 may reflect an adaptive or associated anatomical change in Chiari + SM cases [6].

In females, weak but significant correlations were found between tonsillar herniation and cerebellar circumference (r = 0.372, *p* = 0.001) as well as cerebellum/posterior fossa ratio (r = 0.359, *p* = 0.001). The absence of similar correlations in males suggests that sex-specific cranial volume differences may play a role [29].

In the CM-1 with SM group, SM lesions were predominantly located in the cervical region. The literature also reports a higher prevalence of SM in the cervical region among Chiari 1 cases [30]. This finding suggests a possible association between SM accompanying CM-1 and its effects on CSF flow in the cervical spinal canal. Similarly, in individuals with idiopathic SM, lesions were most frequently localized in the cervical region, indicating that upper spinal segments may be affected even in cases without accompanying Chiari malformation. However, there is no consensus in the literature on this matter. While some studies report a predominance of thoracic localization [10], others emphasize the cervical region [31]. These discrepancies may arise from methodological differences such as patient selection criteria, imaging techniques, and lesion classification.

Saygı et al. reported that posterior fossa narrowing is more pronounced in CM-1 patients with SM and that cervical spinal canal narrowing at the C1–C4 and C1–C7 levels may also be contributory. They further proposed that both morphometric measurements and structural configurations must be considered [32]. In our study, no significant difference was observed between the Chiari and idiopathic groups in terms of posterior fossa area, suggesting that posterior fossa anatomy plays a fundamental role in the pathophysiology of SM.

In the study conducted by Bogdanov and colleagues, the craniospinal MRI of 17 patients with idiopathic cervical SM, 17 patients with SM associated with Chiari Type I, and 32 healthy controls were comparatively analyzed. Structural parameters, particularly posterior fossa morphometry and brainstem position, were evaluated; CSF pathways and SM cavities were examined in detail using RARE-MR hydrography. The study findings demonstrated that morphometric changes such as a reduced posterior fossa height, altered Boogaard angle, and the narrowing of CSF flow pathways play a significant role in the development of SM [33]. Consistent with our findings, the study emphasized that posterior fossa narrowing and restricted CSF circulation may be associated with SM even in cases without tonsillar herniation.

Our findings indicate that morphometric measurements of the posterior fossa and cerebellar structures have important clinical applications not only in elucidating etiology but also in treatment planning. In symptomatic idiopathic SM cases, interventions solely targeting the syrinx cavity, such as intramedullary shunting or aspiration, provide limited therapeutic success as they do not address the underlying etiological factors. Conversely, studies have demonstrated that surgical decompression to relieve structural constrictions and compression within the posterior fossa may be an effective treatment approach for this patient group [8,34].

Currently, advances in artificial intelligence and deep learning algorithms enable the highly accurate automatic calculation of critical anatomical parameters from MRI scans, including posterior fossa volume, cerebellar ratios, and foramen magnum diameter [35,36]. These technological developments facilitate more standardized, rapid, and objective radiological assessments of patients diagnosed with idiopathic SM. Accordingly, morphometric analyses may play an increasingly significant role in surgical decision making, particularly in cases with unexplained etiology.

## 5. Conclusions

This study suggests that focusing solely on tonsillar herniation may not be sufficient for diagnosis and treatment planning in CM-I and SM cases, and that structural parameters such as posterior fossa and cerebellar volumes may provide additional diagnostic value. Moreover, certain cranial morphometric differences were observed in individuals diagnosed with SM but without tonsillar descent, indicating that these measurements could potentially serve as biomarkers in differential diagnosis.

Measurements of posterior fossa area, cerebellar dimensions, and their respective ratios provide valuable insights into the structural background of SM. Furthermore, the consideration of gender-based anatomical differences is essential for a personalized approach.

The limitations of this study include its retrospective design, possible measurement biases due to imaging quality, and small subgroup sample sizes. One notable limitation is the unequal sample sizes across groups, particularly the smaller number of patients in the idiopathic SM group. This discrepancy may reduce the statistical power and increase the likelihood of Type II error in between-group comparisons. Although non-parametric methods were applied where appropriate, findings involving smaller subgroups should be interpreted with caution. Future studies with larger and more balanced samples are warranted to validate these results.

Future prospective, multicenter studies with larger cohorts and advanced CSF flow analyses are needed to better differentiate between CM-0 and idiopathic SM. Additionally, the integration of 3D volumetric analysis techniques may improve the accuracy of morphometric evaluations.

In conclusion, the detailed assessment of cranial morphometric structures may serve as a valuable clinical tool for diagnosis, prognosis, and treatment planning in SM.

## Figures and Tables

**Table 1:** Descriptive statistics (mean ± SD) of cranial morphometric measurements in female participants, grouped by diagnosis: healthy (*n* = 93), Chiari (*n* = 54), Chiari with SM (*n* = 25), and idiopathic SM (*n* = 30). Measurements are categorized into perimeter, area, and ratio-based parameters. Data reflect the mean anatomical characteristics within each subgroup.

Measurement	Healthy (Mean ± SD)	Chiari (Mean ± SD)	Chiari + SM (Mean ± SD)	Idiopathic SM (Mean ± SD)
Foramen Magnum Diameter	34.19 ± 2.78	34.63 ± 4.77	35.29 ± 4.65	33.82 ± 3.02
Intracranial Perimeter	466.74 ± 15.19	465.59 ± 19.83	470.42 ± 17.34	462.73 ± 12.93
Posterior Fossa Perimeter	235.93 ± 13.70	215.68 ± 9.36	218.40 ± 10.76	217.82 ± 9.21
Cerebellum Perimeter	162.22 ± 14.91	170.69 ± 13.70	171.66 ± 13.93	159.49 ± 17.25
Intracranial Area	14,230.10 ± 874.84	14,207.31 ± 1133.66	14,480.87 ± 1124.79	14,042.73 ± 889.31
Posterior Fossa Area	3025.94 ± 287.07	2748.22 ± 235.44	2912.37 ± 254.14	2824.09 ± 185.54
Cerebellum Area	1242.60 ± 241.88	1421.41 ± 171.25	1450.79 ± 214.13	1223.66 ± 187.90
Cerebellum/Fossa. Ratio	0.423 ± 0.199	0.517 ± 0.042	0.484 ± 0.103	0.434 ± 0.064
P.Fos/int. Cr. Ratio	0.213 ± 0.018	0.194 ± 0.017	0.198 ± 0.016	0.202 ± 0.016
Cerebellum/int. Cr. Ratio	0.088 ± 0.017	0.100 ± 0.012	0.100 ± 0.013	0.087 ± 0.012

Fossa: Posterior Fossa Area, int. Cr.: Intracranial Area.

**Table 2:** Descriptive statistics (mean ± SD) of cranial morphometric measurements in male participants, categorized into perimeter, area, and ratio-based parameters. Values are presented for four diagnostic subgroups: healthy (*n* = 63), Chiari (*n* = 25), Chiari with SM (*n* = 14), and idiopathic SM (*n* = 24).

Measurement	Healthy (Mean ± SD)	Chiari (Mean ± SD)	Chiari + SM (Mean ± SD)	Idiopathic SM (Mean ± SD)
Foramen Magnum Diameter	36.02 ± 2.70	35.38 ± 3.76	37.61 ± 4.91	34.41 ± 4.06
Intracranial Perimeter	487.22 ± 16.20	487.45 ± 18.54	484.16 ± 25.01	479.25 ± 42.49
Posterior Fossa Perimeter	239.82 ± 13.83	223.51 ± 13.38	224.97 ± 10.26	226.88 ± 15.31
Cerebellum Perimeter	165.15 ± 12.66	172.02 ± 14.64	179.86 ± 16.50	164.48 ± 13.62
Intracranial Area	15,238.88 ± 1051.16	15,385.07 ± 1322.68	14,553.84 ± 4049.92	15,153.08 ± 1150.84
Posterior Fossa Area	3231.17 ± 465.48	2971.29 ± 300.12	2978.95 ± 251.44	3065.40 ± 217.21
Cerebellum Area	1286.03 ± 130.95	1463.55 ± 168.59	1441.55 ± 139.99	1328.27 ± 188.41
Cerebellum/Fossa Ratio	0.474 ± 0.645	0.494 ± 0.052	0.485 ± 0.041	0.434 ± 0.054
Fossa/int. Cr. Ratio	0.212 ± 0.030	0.193 ± 0.012	0.196 ± 0.018	0.203 ± 0.015
Cerebellum/int. Cr Ratio	0.085 ± 0.010	0.095 ± 0.008	0.094 ± 0.010	0.088 ± 0.012

Fossa: Posterior Fossa Area, int. Cr.: Intracranial Area.

## Data Availability

The data that support the findings of this study are available from the corresponding author upon reasonable request. Due to ethical and privacy restrictions, the data are not publicly available.

## Abbreviations

The following abbreviations are used in this manuscript:

Fossa	Posterior Fossa Area
Int. Cr.	Intracranial area
SM	Syringomyelia
CM-1	Chiari Type 1
CM-0	Chiari Type 0
CSF	Cerebrospinal fluid
